# Shorter telomere length predicts poor antidepressant response and poorer cardiometabolic indices in major depression

**DOI:** 10.1038/s41598-023-35912-z

**Published:** 2023-06-23

**Authors:** Ryan Rampersaud, Gwyneth W. Y. Wu, Victor I. Reus, Jue Lin, Elizabeth H. Blackburn, Elissa S. Epel, Christina M. Hough, Synthia H. Mellon, Owen M. Wolkowitz

**Affiliations:** 1 Weill Institute for Neurosciences and Department of Psychiatry and Behavioral Sciences, University of California San Francisco (UCSF) School of Medicine, San Francisco, CA USA; 2 Department of Biochemistry and Biophysics, University of California San Francisco (UCSF) School of Medicine, San Francisco, CA USA; 3 Department of OB-GYN and Reproductive Sciences, UCSF School of Medicine, San Francisco, CA USA; 4 Present Address: Department of Psychology, University of California Los Angeles (UCLA), Los Angeles, CA USA

**Keywords:** Telomeres, Biomarkers, Depression

## Abstract

Telomere length (TL) is a marker of biological aging, and shorter telomeres have been associated with several medical and psychiatric disorders, including cardiometabolic dysregulation and Major Depressive Disorder (MDD). In addition, studies have shown shorter TL to be associated with poorer response to certain psychotropic medications, and our previous work suggested shorter TL and higher telomerase activity (TA) predicts poorer response to Selective Serotonin Reuptake Inhibitor (SSRI) treatment. Using a new group of unmedicated medically healthy individuals with MDD (n = 48), we sought to replicate our prior findings demonstrating that peripheral blood mononuclear cell (PBMC) TL and TA predict response to SSRI treatment and to identify associations between TL and TA with biological stress mediators and cardiometabolic risk indices. Our results demonstrate that longer pre-treatment TL was associated with better response to SSRI treatment (*β* = .407 *p* = .007). Additionally, we observed that TL had a negative relationship with allostatic load (*β* = − .320 *p* = .017) and a cardiometabolic risk score (β = − .300 *p* = .025). Our results suggest that PBMC TL reflects, in part, the cumulative effects of physiological stress and cardiovascular risk in MDD and may be a biomarker for predicting SSRI response.

## Introduction

Major Depressive Disorder (MDD) affects 5.0% of adults globally (WHO) and has a lifetime prevalence of 15%^1^. In addition to the development of debilitating psychiatric symptoms, MDD is also known to be associated with diseases of aging (including diabetes^2^, cardiovascular disease^3^, obesity^4^) as well as premature mortality^5^. The mechanisms linking MDD to this increased risk have not been fully elucidated, but several studies suggest that depression may be associated with dysregulated physiological stress systems and metabolic dysregulation^6^. Importantly, several studies suggest that dysregulation in such systems is positively correlated with accelerated biological aging^7^. That is, an individual with MDD may experience dysregulation of several biological systems, above and beyond what would be expected from chronological aging, and these alterations may contribute to accelerated biological aging. While several metrics of biological aging have been used to demonstrate this link,^8,9^ telomere length (TL) has been extensively used to index accelerated aging in MDD^10–12^ and other chronic somatic diseases, namely cardiovascular disease^13–17^, diabetes^18,19^ as well as premature mortality in a meta-analysis^20^.

Telomeres are specialized nucleoprotein structures located at the end of chromosomes that are composed of repetitive sequences of non-coding DNA and associated proteins. These telomeric DNA repeat tracts are often shortened with each cell division which, if left unchecked, may lead to cell senescence and death^21,22^ and ultimately contribute to disease^23^. In addition to this replication-induced shortening, DNA damage and genetic, psychosocial, environmental, and behavioral factors such as psychological stress, diet/obesity, and smoking can accelerate the erosion of telomeres leading to premature cellular senescence and death. At a cellular level, exposure to inflammation and oxidative stress also contributes to this process^24–26^. Telomere shortening can be counteracted by telomerase which, in highly regulated ways, adds telomeric repeats to the ends of chromosomes^27^. These endogenous and exogenous variables interact to determine the rate of telomere erosion.

While numerous studies have linked MDD with shortened telomeres^9–11,24,28^, results are not consistent, with some reports demonstrating no differences between MDD and healthy controls^24,29,30^. Rather than being associated with MDD status, some data suggest that shortened telomeres may actually reflect duration of exposure to depressive symptoms^24^, the severity of depressive symptoms^10^, or stress-related physiological or metabolic dysregulation including metabolic syndrome, inflammation and/or oxidative stress^7,12,24,31–34^. For example, studies in non-depressed individuals have demonstrated that the duration/intensity of exposure to chronic stress is significantly related to telomere length^33^. Further, rather than being associated with the categorical diagnosis of MDD, TL may be associated with specific symptoms frequently associated with MDD such as anxiety^35–37^, and this comorbid symptom has been inadequately studied in relation to TL.

Preliminary work from our group demonstrated that longer telomere length predicted better response (defined as ≥ 50% reduction in the Hamilton Depression Rating Scale over an eight-week course of treatment) to Selective Serotonin Reuptake Inhibitors (SSRIs)^38^. That study was the first to report the use of TL as a novel predictor of response to antidepressant medication and was consistent with other studies that subsequently reported that shorter TL was associated with poorer response to certain psychiatric medications, including antipsychotics in schizophrenia^39,40^, adjunctive pioglitazone in MDD^41^, and lithium among patients with bipolar disorder^42^. The search for biomarkers of depression and treatment outcomes is ongoing and, to our knowledge, there have been no additional studies that have prospectively assessed the utility of TL as a predictor of antidepressant treatment response. Other preliminary work from our group found that relatively higher TA levels predicted poorer response to SSRIs in MDD^43^, which was replicated in a more recent study^44^.

In this study, utilizing a new cohort of well-phenotyped, medically healthy, unmedicated depressed participants, we sought to replicate previous findings linking pre-treatment TL to antidepressant response after 8 weeks of treatment with SSRIs and to characterize the relationship of TL and TA to dimensions of MDD severity, including associated symptoms of anxiety, which may bear a relationship with telomere shortening^37^. Additionally, we sought to examine the relationship of TL and TA to biological stress mediators, as well as markers of cardiometabolic risk, which are associated with both TL and TA in depressed individuals. The particular physiological/metabolic indices we assessed included Allostatic Load, Cardiometabolic Risk Score, and Triglyceride Index (TyG), each of which may be associated with TL and/or TA^45–47^.

## Results

### Demographics

The demographic and clinical characteristics of the sample are summarized in Table 1.

**Table 1:** Participant characteristics.

	MDD (n = 48)	Non-responders (n = 22)	Responders (n = 10)	NR versus R *p* value
*Sociodemographic characteristics*				
Age, years	34.9 (10.7)	37.1 (11.7)	35.8 (8.78)	0.732
Sex, female n (%)	26 (54.2%)	11 (50.0%)	6 (60.0%)	χ^2^ = 0.020 * p* = 0.886
Race/ethnicity, n (%)				
White	22 (45.8%)	13 (59.1%)	5 (50.0%)	χ^2^ = 2.004 * p* = 0.735
Black	4(8.3%)	1 (4.55%)	1 (10.0%)	
Asian	12(25%)	5 (22.7%)	1 (10.0%)	
More than one	6(12.5%)	2 (9.09%)	2 (20.0%)	
Other	4(8.3%)	1 (4.55%)	1 (10.0%)	
Years of education (n = 47)	16.0 (2.3) n = 47	15.8 (2.43)	16.3 (2.67)	0.602
*Lifestyle factors*				
Body mass index	25.2 (4.1)	25.3 (4.07)	24.7 (5.02)	0.778
Smoking status, n (%)				
Never	31 (64.6%)	15 (68.1%)	4 (40%)	χ^2^ = 3.0275 * p* = 0.220
Former	13 (27.1%)	6 (27.3%)	4 (40%)	
Current	4 (8.3%)	1 (4.6%)	2 (20%)	
HgbA1c	5.3 (0.38)	5.30 (0.35)	5.12 (0.48)	0.307
MAP	86.6 (9.8)	88.8 (10.1)	83.6 (7.80)	0.125
Triglyceride (TyG) index (n = 45)	4.44 (0.29)	4.43 (0.31)	4.44 (0.39)	0.967
Cardiometabolic Risk (CR) score	4.05 (0.11)	4.07 (0.10)	4.01 (0.13)	0.275
Allostatic Load Score (n = 45)	3 (1.71)	3.20 (1.67)	2.70 (2.16)	0.531
*Symptoms*				
Hamilton Depression Rating Scale (HDRS)	19.4 (3.4)	19.4 (2.89)	18.3 (3.62)	0.408
Perceived stress scale (PSS) (n = 45)	26.0 (5.7)	25.2 (5.63)	26.7 (6.41)	0.529
Duration of depression (months) (n = 42)	190.6 (162)	226 (172)	152 (143)	0.329
*Telomere/telomerase variables*				
T/S ratio	0.96 (0.18)	0.89 (0.16)	1.04 (0.10)	**0.005**
Telomerase activity (n = 47)	11.5 (12.4)	14.4 (16.7)	7.36 (4.09)	0.375

Demographics calculated using only available data. Sample sizes are indicated in parentheses where missing values exist. Missing data (except for HDRS, PSS, and duration of depression) were imputed with the mean for subsequent analyses.

Significant values are in bold.

### Telomere length associations with depressive symptomatology

We observed no significant relationship between TL and the estimated lifetime duration of MDD (*β* = 0.069 *p* = 0.683) or baseline Hamilton Depression Rating Score (HDRS; *β* = − 0.052 *p* = 0.706) after adjusting for age. We also found that the subjective experience of psychological stress over the past month, as measured by the Perceived Stress Scale, was not significantly associated with TL (*β* = − 0.0007 *p* = 0.996). In an exploratory analysis, given a prior report^48^ suggesting that anxiety (and not depressive) symptoms in MDD were associated with elevated oxidative stress (believed to be an important mediator of telomere shortening^49^), as well as reports that anxiety symptoms may bear an even stronger relationship to TL shortening than depression symptoms themselves^36^, we examined the relationship between age adjusted TL with low/high anxiety and low/high depressed mood. We observed that depressed participants with low anxiety had significantly longer age-adjusted TL (0.090 ± 0.179) compared to those with high anxiety (− 0.045 ± 0.133; *t*_46_ = 2.9528 *p* = 0.005, *Cohen’s* *d* = 0.90). Interestingly, we observed no differences in TL in those with low depressed mood (− 0.007 ± 0.132) compared to those with high depressed mood (0.003 ± 0.174; *t*_46_ = − 0.190 *p* = 0.850, Cohen’s *d* = 0.06; Supplementary Fig. 1). With regards to TA, we observed no significant associations between pre-treatment TA and depression severity or estimated lifetime duration of depression (all *p* > 0.05). Additionally, no differences in age-adjusted TA between the low and high anxiety group (*t*_46_ = − 0.334 *p* = 0.740, Cohen’s *d* = 0.10) or between the low and high depressed mood group (*t*_46_ = 0.729 *p* = 0.470, Cohen’s *d* = 0.23) were observed.

### Pre-treatment telomere length and telomerase activity predicts antidepressant response

**Figure 1:**
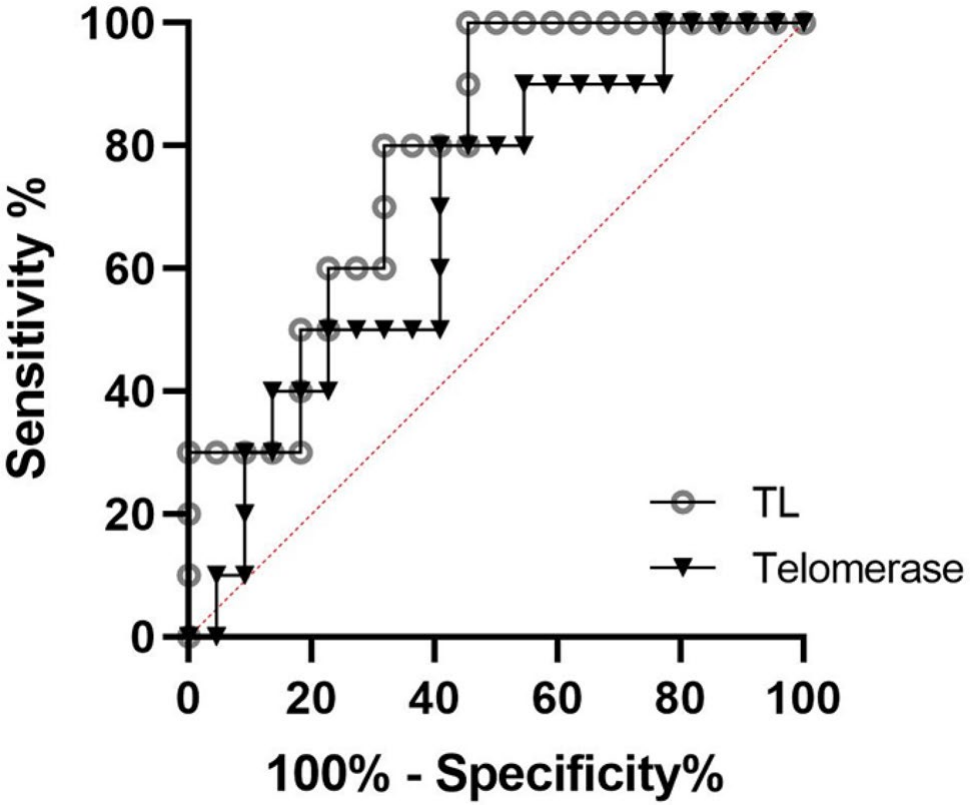
ROC curves of age-adjusted TL and TA in antidepressant response. Diagnostic accuracy of pre-treatment TL (PBMC T/S ratio) and TA predicting Responder status: AUC = 78.6% and AUC = 68.6%, respectively. Data represents age adjusted values for TL and TA.

We observed that TL was positively associated with Responder status after adjusting for age (*β* = 0.407 *p* = 0.007) which remained significant after further adjustment for gender, race, and smoking status (*β* = 0.375 *p* = 0.03). Specifically, antidepressant Non-Responders had shorter pre-treatment age-adjusted TL compared to Responders (− 0.058 ± 0.134 vs. 0.08 ± 0.088; *t*_30_ = − 2.96 *p* = 0.006, Cohen’s *d* = 1.13; Supplementary Fig. 1). We also observed that baseline TL was negatively associated with absolute decrease in HDRS (ΔHDRS) ratings over the treatment period after adjusting for age, although this did not reach statistical significance (*β* = − 0.317 *p* = 0.131). Baseline TL was also negatively associated with decrease in PSS (ΔPSS) over the treatment period after adjusting for age (*β* = − 0.593 *p* = 0.0034; Table 2). A similar pattern of association between age-adjusted TL and TA with ΔHDRS and ΔPSS was observed via Spearman correlation (Supplementary Fig. 3). In a sex-stratified analysis, we found that the relationship between TL and Responder status was only observed for female participants (*p* = 0.037) but not males (*p* = 0.147) while the relationship with ΔPSS was observed in male (*p* = 0.014) but not female (*p* = 0.118) participants (Supplementary Table 1), although these sex differences must be interpreted cautiously due to the small number of subjects when sex-dichotomized. Given that prior studies have suggested race specific differences in TL, we carried out a sensitivity analysis examining TL in the Non-Hispanic White participants and found that greater pre-treatment TL was associated with Response status to antidepressants after adjusting for age (*β* = 0.497 *p* = 0.014), with improvements in subjective stress over the course of an 8 week antidepressant trial (*β* = − 0.615 *p* = 0.001), and with improvements in depression severity, although the latter did not reach statistical significance (*β* = − 0.359 *p* = 0.097). Additionally, given that tobacco use is associated with telomere shortening, we carried out a sensitivity analysis in individuals who have never smoked. Significant relationships were observed between TL and Responder status as well as ΔPSS (Supplementary Table 1) after adjusting for age, indicating that longer TL was associated with better response to treatment and greater decreases in subjective stress over the treatment period, although only the relationship with PSS remained significant after adjustment for additional covariates. Notably, we observed no significant relationship between TL and ΔHDRS in this group. No significant relationship was observed between Responder status and TA (Table 2). Group differences were examined, and Non-responders had higher levels of pre-treatment age-adjusted TA (0.075 ± 0.343) compared to Responders (− 0.093 ± 0.221), although this was not statistically significant (*t*_30_ = 1.4151 *p* = 0.167, Cohen’s *d* = 0.540; Supplementary Fig. 2). However, we did find that lower pre-treatment TA was associated with greater absolute improvement in depression severity (ΔHDRS) after adjusting for age (*β* = 0.390 *p* = 0.05) and improvement in perceived stress (*β* = 0.432 *p* = 0.03; Table 2) after eight weeks of SSRI treatment, which remained significant after adjustment for several covariates. ROC analysis was performed to establish the value of age adjusted TL and TA in predicting response to SSRI antidepressant treatment. Age-adjusted pre-treatment TL performed better than age-adjusted TA in predicting response, with an area under the curve of 78.6% and 68.6% respectively (Fig. 1).

**Table 2:** Associations between telomere length or telomerase activity with response to treatment or change in perceived stress after 8 weeks of antidepressant treatment.

	Telomere length			Telomerase		
	b (95% CI)	β	*p* value	b (95% CI)	β	*p* value
Responder status						
Model 1	0.146 (0.0332, 0.259)	0.435	**0.0129**	− 0.153 (− 0.424, 0.118)	− 0.206	0.257
Model 2	0.137 (0.040, 0.233)	0.407	**0.007**	− 0.172 (− 0.418, 0.073)	− 0.232	0.162
Model 3	0.126 (0.013, 0.239)	0.375	**0.03**	− 0.140 (− 0.428, 0.149)	− 0.188	0.327
Δ HDRS						
Model 1	− 11.12 (− 22.9, 0.700)	− 0.331	0.064	4.61 (− 1.09, 10.31)	0.284	0.109
Model 2	− 10.65 (− 24.7, 3.36)	− 0.317	0.131	6.33 (0.001, 12.66)	0.39	**0.05**
Model 3	− 9.64 (− 26.4, 7.15)	− 0.287	0.247	7.17 (0.012, 14.33)	0.442	**0.05**
Δ PSS						
Model 1	− 23.11 (− 36.0, − 10.2)	− 0.569	**0.001**	4.36 (− 2.56, 11.3)	0.237	0.207
Model 2	− 24.1 (− 39.5, − 8.70)	− 0.593	**0.0034**	7.94 (0.816, 15.1)	0.432	**0.03**
Model 3	− 29.7 (− 44.1, − 15.3)	− 0.731	**0.0003**	8.47 (1.07, 15.9)	0.461	**0.038**

Model 1. Unadjusted.

Model 2. Adjusted for age.

Model 3. Further adjusted for Gender, Race, and Smoking status.

Responder Status: Response defined as > 50% reduction in HDRS score; Positive association indicates longer TL is associated with Response status.

Δ HDRS = Changed in depression severity over the course of 8 weeks of antidepressant treatment.

ΔPSS = Change in Perceived Stress Scale score over the course of 8 weeks of antidepressant treatment.

Significant values are in bold.

In an exploratory analysis, we sought to determine if the combination of TL and TA might further define subgroups with unique clinical and/or biological characteristics, as suggested in other studies^50^, namely response to 8 weeks of SSRI treatment. We noted that group differences existed when comparing groups (short TL/low TA, short TL/high TA, long TL/low TA, low TL/high TA; *F*_(3, 15.058)_ = 4.2134 *p* = 0.024). Post-hoc testing revealed a significant group difference between the short TL/high TA and long TL/low TA group (*p* = 0.003; Bonferroni corrected *p* value < 0.017). (Supplementary Fig. 4).

### Associations of telomere length with biological stress mediators

We assessed the relationship between pre-treatment TL and a biological measure of cumulative physiological stress, Allostatic Load^45^. We observed an inverse relationship between allostatic load and pre-treatment TL after adjustment for age (*β* = − 0.320 *p* = 0.0164), which was no longer statistically significant after adjustment for several covariates as above (*β* = − 0.297 *p* = 0.037; Bonferroni adjusted *p* value is *p* < 0.017; Table 3). Given that inflammation ^32,51^ and oxidative stress ^52–54^ have previously been associated with shortened telomeres, we also examined the relationship between TL and CRP and glutathione peroxidase (GP). We observed an inverse relationship between pre-treatment TL with CRP, although this did not meet statistical significance after adjusting for age (*β* = − 0.224 *p* = 0.104; Table 3). No significant relationship between TL and glutathione peroxidase (GP) was observed (Table 3). A similar pattern of relationship was observed when examining Spearman correlations between these parameters (Supplementary Fig. 5). To determine if shorter baseline TL was a proxy for another variable which was associated with poorer treatment response such as oxidative stress, inflammatory markers or allostatic load, mediation analyses were carried out (data not shown). We found no evidence of statistical mediation between pre-treatment CRP, GP, or allostatic load and improvement in depression severity (all *p*s > 0.10). In an exploratory analysis, we looked at relationships with the individual components that contribute to the allostatic load score. We observed significant inverse relationships between TL and blood pressure parameters, including systolic blood pressure and diastolic blood pressure (*β* = − 0.410 *p* = 0.002 and *β* = − 0.459 *p* = 0.0003; Supplementary Table 2).

**Table 3:** Relationships between telomere length or telomerase and biological mediators.

	Telomere length			Telomerase		
	b (95% CI)	β	*p* value	b (95% CI)	β	*p* value
AL						
Model 1	− 0.037 (− 0.067, − 0.007)	− 0.35	**0.0158**	0.013 (− 0.048, 0.073)	0.062	0.678
Model 2	− 0.034 (− 0.061, − 0.006)	− 0.32	**0.0164**	0.018 (− 0.039, 0.075)	0.09	0.523
Model 3	− 0.031(− 0.061, − 0.002)	− 0.297	0.037	0.014 (− 0.046, 0.075)	0.071	0.635
CRP						
Model 1	− 0.124 (− 0.247, − 0.002)	− 0.289	0.047	0.114 (− 0.126, 0.353)	0.139	0.346
Model 2	− 0.097(− 0.214, 0.021)	− 0.224	0.104	0.173 (− 0.053, 0.399)	0.211	0.131
Model 3	− 0.092 (− 0.220, 0.035)	− 0.214	0.15	0.200 (− 0.045, 0.445)	0.245	0.107
GP						
Model 1	− 0.389 (− 1.10, 0.326)	− 0.159	0.279	− 1.27 (− 2.59, 0.048)	− 0.275	0.059
Model 2	− 0.311 (− 0.977, 0.356)	− 0.127	0.353	− 1.14 (− 2.39, 0.110)	− 0.247	0.073
Model 3	− 0.235 (− 0.915, 0.445)	− 0.096	0.488	− 1.37 (− 2.62, − 0.120)	− 0.297	0.033

Model 1. Unadjusted for age.

Model 2. Adjusted for age.

Model 3. Further adjusted for Gender, Race, and Smoking status.

AL = Allostatic Load; GP = Glutathione Peroxidase; CRP = C-reactive protein.

n = 47 for correlation between with AL due to exclusion.

*Bonferroni-corrected *p* value for significance was *p* = 0.017 (0.05/3).

Significant values are in bold.

### Associations between telomere length and cardiometabolic dysregulation

Given that telomere shortening may be a risk factor for age-related pathology including metabolic dysregulation^55–57^ and cardiovascular disease^16,18,58–60^, we examined associations between TL and markers of insulin resistance (Triglyceride-Glucose index [TyG index]) as well as a marker of cardiovascular risk (cardiometabolic risk [CR] score^61^). We only observed a significant inverse relationship between TL and the CR score after adjusting for age (*β* = − 0.300 *p* = 0.025), which was no longer statistically significant after adjustment for additional covariates (*β* = − 0.269 *p* = 0.06). In an exploratory analysis, we looked at relationships with the individual components of these composite scores. We observed significant inverse relationships between TL and mean arterial pressure (*β* = − 0.465 *p* = 0.0003), which remained significant after adjustment for several covariates (Supplementary Table 3).

### Associations between telomerase, biological stress mediators, and cardiometabolic dysregulation

TA showed no significant associations with allostatic load or CRP (Bonferroni adjusted *p* value is *p* < 0.017; Table 3). An inverse relationship was observed between TA and GP after adjusting for age, although this did not reach statistical significance (*β* = − 0.247 *p* = 0.073). Additionally, no significant associations of TA with the TyG index or CR score were observed (*p* > 0.10; Table 4).

**Table 4:** Relationships between telomere length or telomerase and cardiometabolic indices.

	Telomere length			Telomerase		
	b (95% CI)	β	*p* value	b (95% CI)	β	*p* value
TyG Index						
Model 1	− 0.105 (− 0.288, 0.077)	− 0.171	0.25	0.147 (− 0.204, 0.498)	0.125	0.404
Model 2	− 0.086 (− 0.254, 0.082)	− 0.14	0.308	0.181 (− 0.149, 0.511)	0.154	0.274
Model 3	− 0.128 (− 0.314, 0.058)	− 0.208	0.17	0.170 (− 0.198, 0.538)	0.144	0.355
CR score						
Model 1	− 0.511 (− 0.978, − 0.043)	− 0.309	**0.033**	0.391 (− 0.532, 1.31)	0.125	0.398
Model 2	− 0.497 (− 0.928, − 0.066)	− 0.3	**0.025**	0.416 (− 0.452, 1.28)	0.133	0.34
Model 3	− 0.445 (− 0.909, 0.019)	− 0.269	0.06	0.389 (− 0.544, 1.32)	0.124	0.405

Model 1. Unadjusted.

Model 2. Adjusted for age.

Model 3. Further adjusted for Gender, Race, and Smoking status.

CR score = Cardiometabolic Risk score; TyG index = Triglyceride index.

n = 47 for correlation between with TyG index due to exclusion.

*Bonferroni-corrected *p* value for significance was *p* = 0.025 (0.05/2).

Significant values are in bold.

## Discussion

In this study, we set out to replicate the findings of our prior studies examining the relationship between depression and antidepressant treatment with TL and TA in a new cohort of unmedicated medically healthy individuals with MDD. Our most important findings in this new cohort of MDD individuals were (i) no significant associations between pre-treatment TL or TA with pretreatment depression severity/chronicity; (ii) significant negative association between pre-treatment TL and pretreatment anxiety ratings; (iii) pre-treatment TL predicted SSRI antidepressant responsiveness, with longer TL associated with greater likelihood of achieving Responder status after 8 weeks of SSRI treatment; and (iv) an inverse association between pretreatment TL and allostatic load as well as cardiovascular disease risk factors.

Our findings examining the association between pre-treatment TL and SSRI response are consistent with previously published preliminary results^38^ in a similar cohort of depressed individuals studied in a similar treatment paradigm and suggests that TL has moderate accuracy to predict which individuals will respond to an SSRI trial. We observed that, in base pair (bp) units, Non-Responders had a mean pre-treatment TL of 5247 ± 384 bp while Responders had a mean pre-treatment TL of 5780 ± 249 bp. A prior study by Pisanu et al.^62^ found no association between antidepressant treatment responsiveness and TL. Those results are distinct from our own, and while the reasons for these differences are not clear, it is possible they may be related to the fact that their study compared only “treatment-resistant depression” (defined as historical failure to respond to two or more adequate antidepressant trials over variable lengths of time) with historical antidepressant responders. These designations were made retrospectively, and response status was determined based on various antidepressant classes. In contrast, our study prospectively assessed TL and the response to a single 8-week antidepressant trial and used SSRIs exclusively, which may represent a population and time course that is distinct from the historical responders identified in the Pisanu study.

With regards to TA, while we found no relationship between pre-treatment levels of TA and antidepressant Responder status, our results raise the possibility that lower baseline TA may be associated with greater absolute improvements in depression severity as well as perceived stress after 8 weeks of SSRI treatment. This pattern of association is consistent with our prior report of relatively low PBMC TA predicting superior SSRI response in a separate cohort of depressed participants^43^ and with a more recent report showing that responders to escitalopram treatment had lower pre-treatment TA compared to non-responders^44^. While we observed no significant relationship between TA and CRP via linear regression, we did observe TA to be positively correlated with this non-specific marker of inflammation ($\uprho$** =** 0.29, *p* = 0.046). While speculative, this raises the possibility that greater TA may index an inflammatory state, which is independently associated with poor antidepressant response^63,64^. The relationship between chronic inflammation^65,66^ and TA has been shown previously and may be due to telomerase activation in activated lymphocytes^64,65^. Future studies are needed to better understand the putative relationship between telomerase and response to psychotropic medications^67^.

Consistent with its relationship to SSRI antidepressant treatment response in MDD observed in this and our prior study, several studies suggest that TL may have an association to response to several other medications in a variety of psychiatric diseases. A previous study by Rasgon et al.^41^ found that longer TL predicted better response to adjunctive pioglitazone, added to standard antidepressant treatment, in inpatients with unremitted MDD. Extending into other mood disorders, Martinsson et al.^42^ found, that TL was longer in individuals with bipolar disorder who had responded to lithium treatment compared to those had not responded. These individuals were stable on a lithium dose for several months prior to TL assessment, making it difficult to determine if pre-treatment TL predicted response or if TL was increased by lithium treatment to a greater degree in those who responded. Additionally, two prior studies^39,40^ found that shorter TL was associated with poor response to antipsychotic treatment in individuals with schizophrenia. Given these studies, it is possible that TL might be relevant in a variety of psychiatric diseases and psychotropic medications to identify those who might show a positive response to interventions.

The mechanisms linking shortened TL with poorer antidepressant response are not fully understood, but it is possible that short TL may be a proxy for other variables (such as chronic inflammation, oxidative stress^68^, metabolic dysregulation^69^, HPA axis dysfunction) that may be more directly related to treatment response. While not an exhaustive investigation, none of the inflammatory mediators or oxidative stress markers investigated in this study statistically mediated the association between telomere length and change in depression severity or change in perceived stress over the course of treatment. While this does not preclude some of these mediators as being contributing factors, it also raises the possibility that short telomeres may be a proxy for some other factor (not assessed in this study, e.g., early childhood trauma) which is associated with shortened telomeres^70–72^ and poor response to psychotropic medications^73^. Additionally, shorter peripheral TL may relate to processes which are occurring within the brain that might contribute to treatment resistance. Specifically, shorter PBMC TL may be associated with smaller hippocampal volumes^74,75^ which has been related to treatment resistance in animal models^76^ as well as human studies^77^. Given that TL may reflect the cumulative effects of a variety of exposures, biological disruptions, as well as genetic factors, it is possible that a combination of these factors may contribute to treatment resistance. Unfortunately, we could not examine the relative contributions of all the possible factors which may relate to TL. Future studies should examine this phenomenon in larger samples and focus on disentangling the relative contributions of each of these factors to the relationship between TL and antidepressant treatment resistance.

TL was not associated with the duration or severity of the depression and was unrelated to the subjective experience of recent stress. These findings, too, are consistent with some prior studies^29,62,78^ but differ from others^24,28^. These inconsistent findings throughout the literature^29,41,78,79^ may reflect age of the study population, differences in depression duration, presence of medical co-morbidity, or the existence of biologically distinct subgroups of depressed individuals with unique relationships to TL. For example, we found that TL shortening (but not TA) was most prominent in MDD individuals with elevated markers of physiological dysregulation (i.e., allostatic load), which is discussed further below. Additionally, it is possible that TL shortening may be more or less associated with specific clinical phenotypes (with unique biological alterations). In particular, TL shortening may bear a stronger relationship with the specific symptom of anxiety compared to the specific symptom of depressed mood, as suggested in other reports^36^. This idea is supported by our finding that TL shortening was associated with high levels of psychic anxiety but not high levels of depressed mood. Those individuals with low anxiety had a mean TL of 5743 ± 526 base pairs compared to those with high anxiety (5531 ± 352 bp). While the mechanisms linking anxiety to shorter TL are not clear, prior work from our group demonstrated that anxiety (but not depression) was associated with greater oxidative stress markers^48^. However, we did not observe any associations between the oxidative stress marker assessed here (glutathione peroxidase) and TL. This may be due to the fact that this marker is distinct from those previously assessed^80^. Future studies examine the association of aging metrics with specific symptom domains in addition to MDD as a single diagnostic entity and attempt to link these phenotypes with specific biological disturbances.

While our a priori hypotheses involved TL and TA as isolated measures, we explored whether examining these in tandem might convey additional information. This approach was suggested by several prior reports of shortened TL combined with increased TA in certain diseases states, chronic stress states, or disease risk factors^81,82^ and we examined whether subgroups of depressed individuals existed based on these TL/TA profiles. We observed that depressed individuals with shorter pre-treatment TL combined with higher pre-treatment TA had significantly less improvement in depression severity after 8 weeks of treatment compared with those with longer TL and lower TA. While this analysis is preliminary and needs to be replicated, it raises the possibility that the combination of TL and TA might be informative with regards to prediction of SSRI response and comports with our prior reports in a different MDD cohort^38,43^. The biological underpinnings of this treatment resistance in those with short TL and high TA are uncertain, but this combination may reflect a group with high levels of inflammation^83^ or some other feature which confers treatment resistance. This pattern of short TL/high TA may represent a dysregulated combination^81^ or an unsuccessful compensatory attempt^84^ and has been shown to be associated with poorer health outcomes^85–87^ as well as psychological stress^81^. Considering TL and TA jointly has received little attention in psychiatric illness, and our results suggest this may be a fruitful avenue for future investigations.

The other major finding of this study was that TL was significantly correlated with a physiological measure of cumulative stress exposure (Allostatic Load Score) and cardiometabolic health indices (Cardiometabolic Risk Score). This suggests that even amongst well-screened, medically healthy individuals with MDD, shorter TL is associated with physiological dysregulation, which may portend poorer cardiometabolic outcomes^88–91^ and is driven mainly by the association with alterations in blood pressure. A wealth of studies in non-depressed populations with medical conditions (including hypertension) have demonstrated the association between cardiovascular parameters and telomere length^90,92^. These findings also fit with a prior study in healthy individuals, demonstrating that participants with short TL and high TA demonstrated poor ability of blood pressure to recover after exposure to a stressful mental task^81^ and may be related to oxidative stress, inflammation, endothelial cell dysfunction, or dysregulated stress response systems^93,94^. While numerous studies have demonstrated associations between chronic inflammation or oxidative stress^24,26,95^ and short TL, we did not observe significant associations with a single measure of oxidative stress and only a trend level association with CRP prior to adjustment for age (a non-specific marker of acute inflammation). Notably, we did observe an association between TA and the oxidative stress marker, although this was not significant after adjustment for multiple comparisons. The lack of a finding here does not preclude these from being important mediators of telomere shortening and could be related to the sample size, the level of medical health of our participants, or the assessment of a limited number of inflammatory/oxidative stress markers. Additionally, our study relies on the measurement of peripheral markers without the ability to measure these markers centrally. Despite this, several studies have linked peripheral measures of TL and TA to brain structural changes^75,96,97^ and neurogenesis, which may relate to MDD and treatment response. Future studies should further investigate these associations to link peripheral measures to brain changes that may contribute to MDD and treatment response.

Strengths of the study include our a priori hypotheses based on our previous studies, and while it is a small clinical sample, we replicated our findings of the predictive utility of PBMC TL for the primary outcome of depressive symptoms. We had a highly selected sample that allows us to test biological relationships: The use of medically healthy depressed individuals limits the effects of medical co-morbidity on TL or antidepressant responsiveness. Additionally, our use of medication-free subjects and a prospective study design limit the effects of concurrent psychotropic use on the parameters assessed. Limitations of the study include the use of a modest sample size, requiring replication in larger cohorts to identify individual subject-level contributors to treatment response. Additionally, while we examined associations in medically healthy participants, these results must be extended and validated in more diverse cohorts with a wider range of medical and psychiatric co-morbidity. The short time frame nature of this study is also a limitation, as longitudinal analyses and longer-term outcomes could determine if pre-treatment TL also predicts longer-term response and remission and would be better equipped to understand the role of biological mediators in erosion of telomeres. Similarly, while we found that shorter TL was significantly associated with measures of physiological dysregulation and cardiometabolic risk factors in this population, we do not have long-term longitudinal data that actually show increased incidence of somatic illness. Additionally, while we studied peripheral leukocyte TL, it is unclear whether this reflects aging in other tissues, and future studies should include assessments of other cell types. As we only assessed TL and TA in relationship to 8 weeks of SSRI treatment, it is unknown if the findings extrapolate to other antidepressant classes. Lastly, given that early life stress exposure has been linked to shorter TL^98–102^ and poorer response to antidepressants^73^, these exposures may also be relevant to the assessment of TL and should also be examined in such research.

In conclusion, we demonstrate that PBMC TL may be an easily accessible and measurable biomarker with moderate ability to identify individuals who may respond to SSRI treatment. Furthermore, our results suggest that even among medically healthy individuals with MDD, shortened telomeres are associated with physiological dysregulation that may portend the development of poor cardiometabolic outcomes. Prospective studies will be needed to assess the ability of TL to predict poor health outcomes in individuals with MDD. Currently no reliable biomarkers of antidepressant treatment response exist, although several other studies have identified putative predictors such as oxidative stress markers^68^, metabolic parameters^69^, and circulating hormone levels^103^ and others as possible biomarkers. While TL is a promising biomarker, its predictive power does not yet warrant its use in routine clinical practice, and future studies in larger, more diverse cohorts are needed to confirm these results and to possibly identify an optimal cutoff point for the identification of antidepressant treatment responders. Further, TL and TA may be useful in treatment response prediction in combination with other biomarkers and may help clarify factors that mitigate antidepressant response. The development of reliable biomarkers for prediction of treatment response is of great clinical importance and may facilitate more personalized treatments for this devastating disease.

## Methods

### Ethics statement

This research was approved by the Institutional Review Board (IRB) of the University of California, San Francisco (UCSF), and all participants gave informed consent. This study was carried out in accordance with the Declaration of Helsinki.

### Recruitment procedures and study participants

We present data on forty-eight adult participants that have not previously been reported. Recruitment and study procedures were identical to those used in a previous cohort^38^. These participants were diagnosed with MDD, without psychotic symptoms, according to the Structured Clinical Interview for DSM-IV-TR Axis I Disorders (SCID)^104^, which was the version in use at the beginning of this study and was verified by clinical interview with a board-certified psychiatrist. Depressive symptomatology was evaluated with the 17-item Hamilton Depression Rating Scale (HDRS), with a current score of ≥ 17 being an inclusion criterion. Depressed participants were excluded for presence of the following: bipolar disorders, psychotic symptoms, history of psychosis outside of a mood episode, any eating disorder or post-traumatic stress disorder (PTSD) within one month of entering the study, and substance abuse or dependence (including alcohol) within six months of entering the study. The participants had no acute illnesses or infections, chronic inflammatory disorders, neurological disorders, or any other major medical condition. All subjects were free of psychotropic medications (except as noted below), including antidepressants, and other potentially interfering medications and had not had any vaccinations for at least 6 weeks prior to enrollment in the study, and none was taking vitamin supplements above the US recommended daily allowances. Short acting sedative-hypnotics were allowed as needed for sleep up to a maximum of 3 times per week, but none within 1 week prior to blood draws and behavioral ratings. Prior to each study visit, all subjects had to pass a urine toxicology screen for drugs of abuse (marijuana, cocaine, amphetamines, phencyclidine, opiates, methamphetamine, tricyclic antidepressants, and barbiturates), and a negative urine test for pregnancy for women of child-bearing potential was required. Of the forty-nine participants with MDD enrolled at baseline, thirty-four completed eight weeks of treatment, described below. Five subjects were enrolled solely for the baseline visit only, five withdrew due to Selective Serotonin Reuptake Inhibitors (SSRI) side effects, two were lost to follow up, one withdrew for personal reasons, one withdrew after the baseline visit due to desire to no longer be in the study, one was removed after their baseline visit due to difficulty complying with study procedures.

### Procedures

The subjects were admitted as outpatients to the University of California San Francisco (UCSF) Clinical and Translational Science Institute between the hours of 08:00 h and 11:00 h, having fasted (except water) since 22:00 h the night before. They were instructed to sit quietly and relax for 25–45 min before blood samples were obtained for TL and TA assessment and routine clinical laboratory assessments were made to assess health. Telomere length and TA measurements (described below) were obtained from peripheral blood mononuclear cells (PBMCs) which were obtained from fresh whole blood by Ficoll separation, as described previously^43^. Following this baseline visit, 32 MDD participants completed 8 weeks of open-label outpatient treatment with an SSRI antidepressant, as determined to be clinically appropriate by the study psychiatrist. The prescriber and raters were blinded to measures of TL and TA. Assessments of outpatient compliance with the medication regimen, as well as clinical evaluations and assessments of drug tolerability, were made by a telephone check-in at the end of week 1 and in-person check in at the end of week 4 and week 8, at which time pill counts were performed and plasma SSRI concentrations were assayed. Plasma antidepressant concentrations were in the reported clinical range for that antidepressant, suggesting excellent compliance. To limit the range of treatments, participants with depression were treated with one of four SSRIs: fluoxetine (n = 6), sertraline (n = 13), citalopram (n = 4), and escitalopram (n = 11). The decision regarding the specific SSRI prescribed was made based on clinical grounds, such as medical history, family history, and potential side effects.

### Depression and perceived stress measures

The severity of depressive symptoms at baseline and week 8 was rated using the HDRS^105^. We also assessed for subjective experience of stress over the last month using the Perceived Stress Scale (PSS)^106^ at each timepoint. In order to assess the effects of “pure anxiety” and “pure depression” separately, we chose to utilize a single item on the HDRS to categorize participants into low and high anxiety and depression categories, similar to a prior study from our group^48^. Briefly, to assess “pure anxiety”, we used the “psychic anxiety” item of the HDRS which assesses symptoms such as irritability, tension, worry, and fear (as opposed to the somatic anxiety item of the HDRS which assesses physical symptoms associated with anxiety including GI symptoms such as indigestion/diarrhea/belching, cardiovascular symptoms such as palpitations/headaches, and respiratory symptoms such as hyperventilation among others). To assess “pure depression” we utilized the “depressed mood” item of the HDRS. For data analysis purposes, participants were divided, a priori, into low anxiety (score of 0 or 1 out of a possible score of four) and high anxiety (2 or greater out of a possible score of four), based on our prior methods^48^. Additionally, participants were divided into low depression (score of 1 or 2 out of a possible score of four, as this is a core feature of MDD diagnosis) and high depression (score of 3 or greater out of a possible score of four). Lifetime depression chronicity was evaluated using the life history charting methods described by Post et al.^107^, as we have reported previously^24^. Clinical ratings and history-taking were performed blinded to laboratory testing results.

### Measurement of clinical laboratory values

Quest Laboratory (San Jose, CA) analyzed plasma samples to measure C-Reactive Protein (hs-CRP), blood chemistry (basic metabolic panel, hemoglobinA1c, fasting blood glucose, complete blood count), and lipid panel (triglycerides, cholesterol, LDL, HDL, etc.). SSRI concentrations were assayed from plasma by Thomas B. Cooper (Analytical Psychopharmacology Laboratory, Nathan Kline Institute, Orangeburg, NY 10962).

### Measurement of telomere length

Genomic DNA was extracted as one batch from all the peripheral blood mononuclear cells (PBMCs) samples with the QIAamp mini DNA kit (QIAGEN cat# 51106). DNA was quantified by measuring OD260 with a NanoDrop 2000c Spectrophotometer (Nanodrop Products, Wilmington, DE, USA). All samples passed the quality control of OD260/OD280 between 1.7 and 2.0, concentration greater than 10 ng/μl. DNA was stored in − 80 °C and TL assays were performed within 2 weeks of DNA extraction. The TL measurement assay was adapted from the published original method by Cawthon (Cawthon, 2002; Lin et al., 2010). The telomere thermal cycling profile consisted of cycling for T(telomeric) PCR: denature at 96 °C for 1 min, one cycle; denature at 96 °C for 1 s, anneal/extend at 54 °C for 60 s, with fluorescence data collection, 30 cycles; cycling for S (single copy gene) PCR: denature at 96 °C for 1 min, one cycle; denature at 95 °C for 15 s, anneal at 58 °C for 1 s, extend at 72 °C for 20 s, 8 cycles; followed by denature at 96 °C for 1 s, anneal at 58 °C for 1 s, extend at 72 °C for 20 s, hold at 83 °C for 5 s with data collection, 35 cycles.

The primers for the telomere PCR were tel1b [5′-CGGTTT(GTTTGG)5GTT-3′], used at a final concentration of 100 nM, and tel2b [5′-GGCTTG(CCTTAC)5CCT-3′], used at a final concentration of 900 nM. The primers for the single-copy gene (human beta-globin) PCR were hbg1 [5′-GCTTCTGACACAACTGTGTTCACTAGC-3′], used at a final concentration of 300 nM, and hbg2 [5′-CACCAACTTCATCCACGTTCACC-3′], used at a final concentration of 700 nM. The final reaction mix contained 20 mM Tris–HCl, pH 8.4; 50 mM KCl; 200 μM each dNTP; 1% DMSO; 0.4 × Syber Green I; 22 ng E. coli DNA; 0.4 Units of Platinum Taq DNA polymerase (Invitrogen Inc.); approximately 6.6 ng of genomic DNA per 11 µl reaction. Tubes containing 26, 8.75, 2.9, 0.97, 0.324 and 0.108 ng of a reference DNA (human genomic DNA from buffy coat, cat # 11691112001 from Sigma-Aldrich) were included in each PCR run so that the quantity of targeted templates in each research sample was determined relative to the reference DNA sample by the standard curve method. The same reference DNA was used for all PCR runs. Assays were run in triplicate wells on 384-well assay plates in a Roche LightCycler 480 and the concentration of each well was calculated using the absolute quantification method using the LightCycler 480’s software, v 1.5.1. After applying Dixon’s Q test to remove outliners, the average concentrations of T and S from the triplicate wells were used to calculate the T/S ratios. T/S ratio for each sample was measured twice. When the duplicate T/S value and the initial value varied by more than 7%, the sample was run the third time and the two closest values were reported. 15% of these samples were run a third time. The PCR efficiencies for the T and S reactions were 96.1 ± 0.07% and 98.9 ± 0.71% respectively. DNA extraction and TL assays for the entire study were performed using the same lots of reagents. Lab personnel who performed the assays were provided with de-identified samples and were blind to all demographic and clinical data. TL was assayed before the publication of the Telomere Research Network’s recommendation of using intraclass correlation (ICC) of repeat DNA extraction as an assay precision metric, therefore, ICC for this specific study is not available”. However, repeat DNA extraction from 36 PBMC samples processed using the same protocol, assayed using the same qPCR method in the same lab has an ICC of.

0.955 (CI = [0.914, 0.977]). While age-adjusted T/S ratio was used for all analyses, we present TL in units of base pairs (bp) where indicated in the discussion. Raw T/S ratios were converted to bp using the formula b* p* = 3,274 + 2413 × (T/S). These values represent conversion of the raw T/S ratios which have not been adjusted for age. This formula was derived using a set of cultured cell line DNA samples and is an external conversion formula^108^.

### Measurement of telomerase activity

We optimized the telomerase activity assay on the basis of published protocols^109^.

Three concentrations (2500, 5000 and 10,000 cells) were used for Telomeric Repeat Amplification Protocol (TRAP) reactions to ensure that the assay for each sample is in the linear range. The reaction follows the manufacturer’s protocol. Products are separated on 10% polyacrylamide 8 M urea gels, exposed to phosphorimager screen overnight and scanned using a phosphorimager, and quantified using ImageQuant software (GE Healthcare, Piscataway, NJ, USA). In all, 293 T cells are used as positive controls. Telomerase activity is defined as 1 unit = the amount of product from one 293 T cell/10 000 PBMC’s. Details of the telomerase activity assay method can be found in Lin et al.^110^. Measurement of 24 resting PBMC samples on different days produced an inter-assay CV of 6.7%.

### Calculation of allostatic load, cardiometabolic risk score and TyG index

Individual blood levels of allostatic load components were assayed from serum as described by Hough et al.^69^ Allostatic load (AL) was calculated using methodology from the MacArthur Studies of Successful Aging^111^, with the exception of 12-h urinary catecholamine levels, as urine was not analyzed in the present study. Briefly, tertiles of the eight AL components were determined amongst the healthy controls including those in the parent study^69^ and these cutoff values were used to calculate AL components: systolic blood pressure, diastolic blood pressure, waist-hip-ratio, total cholesterol, HDL cholesterol, HgbA1c, serum cortisol, and serum DHEAS. Tertiles for waist-hip-ratio (WHR) were determined separately between men and women, due to differences in waist measurement site and normative standards between men and women. All subjects received one point for each AL factor at or above the upper tertile, with the exception of HDL cholesterol and DHEAS, which received one point for being at or below the lowest tertile. These contributing factors were then summed to create a total AL score ranging from 0 to 8 for each subject. Cardiometabolic risk (CR) score was assessed by combining three biomarkers (Body mass index [BMI], Mean arterial BP [MAP], and Hemoglobin A1c [HgbA1c]), as previously described^61^. (1) BMI score was calculated as weight in kilograms divided by the square of height in meters. (2) Resting diastolic and systolic blood pressure (BP): Subjects were instructed to sit quietly and relax for 25–45 min before vital signs were taken. MAP was calculated according to the following formula: [(systolic BP) + (2 × diastolic BP)]/3. (3) HbA1c was assessed via Quest Laboratories (San Jose). HbA1c provides an indication of average blood glucose concentrations over the preceding 2 to 3 months. No subjects had a HgbA1c at or above 6.5 (suggestive of diabetes). Triglycerides (TG), and fasting blood glucose (FBG) were assessed via Quest Laboratories (San Jose). The Triglycderide (TyG) index was calculated as ln [TG (mg/dL) × FBG (mg/dL)/2], derived from previous studies^112,113^ and has been shown to be superior to homeostasis model assessment insulin resistance (HOMA-IR) index in predicting insulin resistance^114^.

### Glutathione peroxidase assay

Direct measurement of reactive oxygen species (as a measure of oxidative stress) can be difficult as a result of their short life span and reactivity^115^. In order to assess the extent of oxidative stress of participants, we assessed antioxidant status. Specifically, we examined Glutathione peroxidase activity which was measured in duplicate from plasma, using a colorimetric assay according to the instructions from the manufacturer (BioVision, Inc., Milpitas, California, USA). The coefficient of variation was < 10% and LLOQ was 0.5 nmol NADPH/ ml/min. Higher levels of GP activity reflect greater antioxidant potential and thus lower levels of oxidative stress.

### Statistical analysis

The baseline sample included 48 participants with MDD. One subject was missing data for telomerase, three subjects were missing data for allostatic load, three subjects were missing data for glucose, two subjects were missing data for WHR, three were missing data for the Perceived Stress Scale, six subjects were missing data for chronicity of depression. Relationships with depression severity and chronicity at baseline, as well as associations with baseline Perceived Stress Scale (PSS) score were assessed via linear regression. We present the unadjusted model as well as additional models first adjusted for age and subsequently adjusted for other possible covariates including gender, race, and smoking status which may all be associated with telomere length. Analyses were carried out with available data and sample sizes are indicated in the demographic table for these analyses. Of the 48 individuals, 32 were treated longitudinally with one of four SSRI medications. Missing data for telomerase, allostatic load, diastolic blood pressure, BMI, glucose and WHR were imputed with the mean; sensitivity analyses were carried out without those subjects and no results were changed. For analyses involving TyG index, Allostatic Load, glucose, HDL cholesterol, total cholesterol, and triglycerides, one MDD participant was not included as they were not fasting at the time of their blood measures. Data were assessed for normality and the appropriate parametric or non-parametric test was applied. For purposes of characterizing response to anti-depressant treatment, ‘Responders’ were defined as subjects whose week 8 HDRS ratings improved by ≥ 50% relative to baseline, and ‘Non-responders’ as those with lesser degrees of improvement. Delta HDRS (absolute difference between depression severity at week 8 and week 0) was also used to examine relationships between baseline TL and TA with improvements after SSRI treatment.

Group differences (low/high anxiety or low/high depression) were assessed via independent samples t-test for normally distributed variables and Wilcoxon rank sum test was applied for non-normally distributed variables. To assess the relationships between TL and variables of interest, separate linear regression analyses were carried out. A priori covariates of age, gender, race, and smoking status were included in sequentially adjusted models. In the body of the text, we present the results of the age-adjusted linear regression models (Model 2), but present both unadjusted and fully adjusted models in the Tables throughout. Spearman correlations are presented in the Supplementary Data to identify any non-linear relationships between the variables of interest. All tests were 2-tailed with α = 0.05. In our analysis assessing relationship with biological stress markers, we correct for multiple comparisons using Bonferroni correction (0.05/3, *p* value < 0.017). For relationships with cardiometabolic indices, we correct for two tests (CR score and TyG index) using Bonferroni correction (0.05/2, *p* value < 0.025). To identify groups based on the combination of TL and TA, both variables were dichotomized (≤ median and > median) and four groups were created based on these groupings: Short TL + Low TA, Short TL + High TA, Long TL + Low TA, Long TL + High TA. Telomere length + TA group differences in change in depression severity over the course of eight weeks (ΔHDRS) was assessed by ANOVA. Given prior studies suggesting that the shortTL/highTA group represents a state in which the organism is unable to activate telomerase to an extent high enough to counteract the erosion of telomeres^81^, we planned to carry out post-hoc comparisons between this group and all other groups (Bonferroni corrected *p* value is 0.05/3 = 0.017).

We observed a significant negative association of age with TL (r = − 0.47 *p* = 0.009). While some studies have suggested that sex^116^ and ethnicity^117,118^ may have a relationship with TL, these results have not been consistent^119,120^. In our data on this sample, we observed no group differences in TL based on race/ethnicity (*F*_1,43_ = 0.514 *p* = 0.726) or sex (*t*_47_ = 1.04 *p* = 0.305; Males vs. Females: 0.936 ± 0.190 vs. 0.989 ± 0.163), and no significant correlations with body mass index (BMI; r = − 0.06 *p* = 0.686). Thus, for group differences and secondary correlation analyses (but not the linear regression analyses for which age was included in the model), we adjusted for age by utilizing the residuals after regressing on age, and thus values presented may be positive or negative. There was a significant negative correlation between TA and age (r = − 0.360 *p* = 0.013), consistent with previous studies^121^. We observed no group differences in telomerase activity based on race/ethnicity (*F*_1,44_ = 1.303 *p* = 0.284) or sex (*t*_47_ = − 1.7335 *p* = 0.09) in the sample and no significant correlations with BMI (r = − 0.126 *p* = 0.395). Thus, for group differences and secondary correlation analyses (in the Supplementary) we adjusted log transformed telomerase for age by utilizing the residuals after regressing on age, and thus values may be positive or negative.

## Supplementary Information

Supplementary Information.

## Data Availability

The datasets used and/or analyzed during the current study are available from the corresponding author on reasonable request.
