# Supplementary material for: Shorter telomere length predicts poor antidepressant response and poorer cardiometabolic indices in major depression
Source: Sci Rep. 2023 Jun 23;13:10238. doi: 10.1038/s41598-023-35912-z (PMC10290110; doi:10.1038/s41598-023-35912-z)
Supplement: Supplementary file 1 — Supplementary Information. [file 41598_2023_35912_MOESM1_ESM.docx]

**
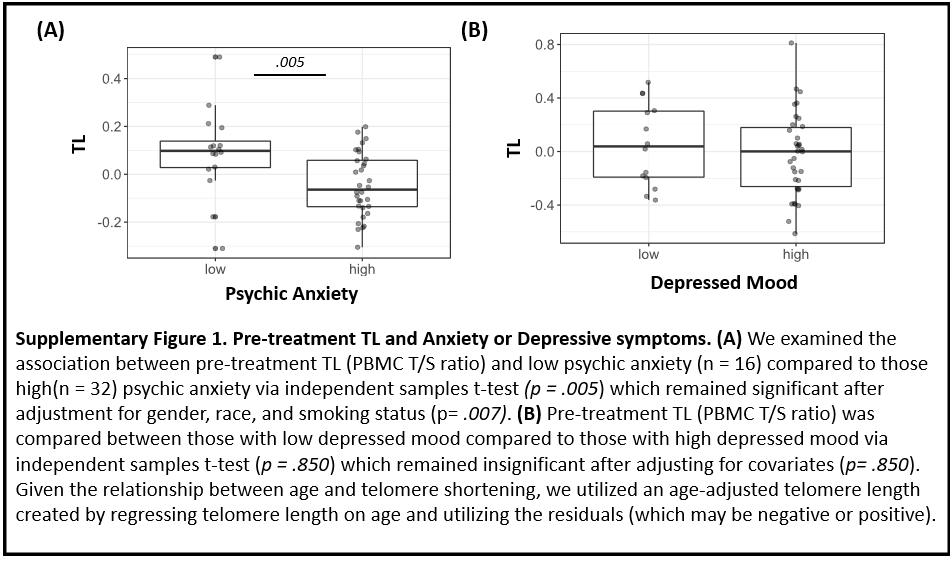
Supplementary Figure 1**

**
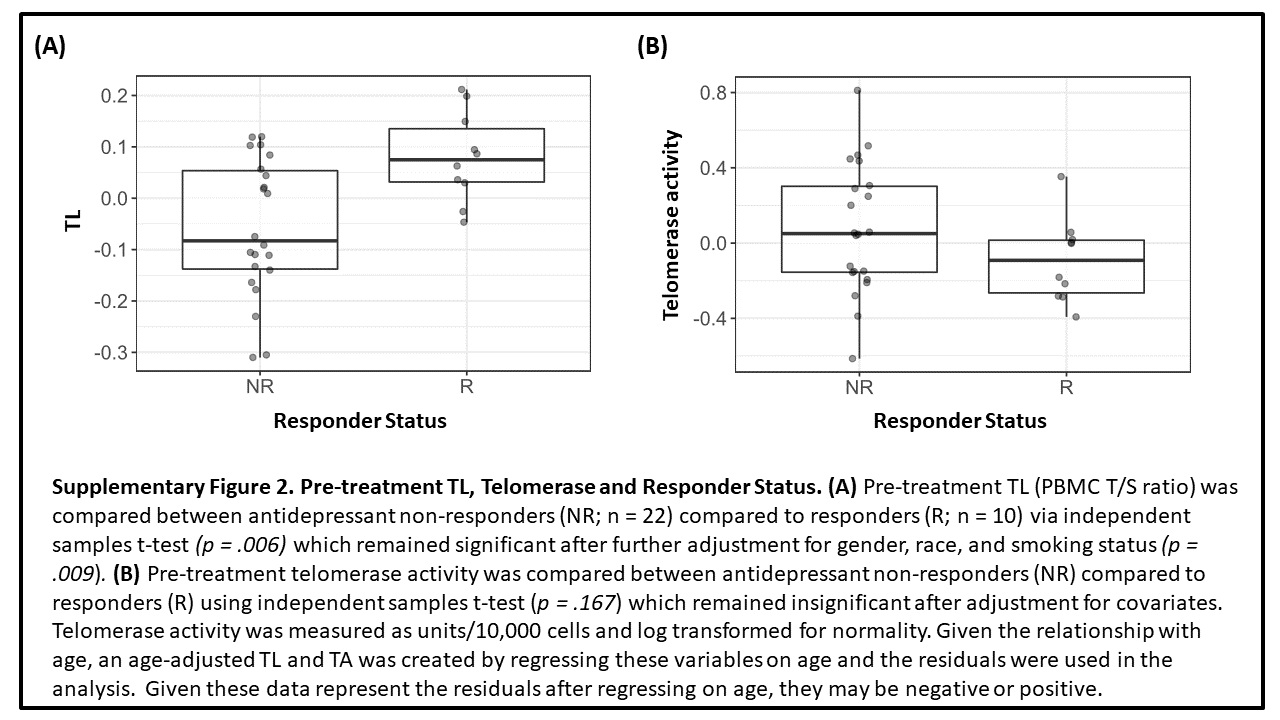
Supplementary Figure 2**

**
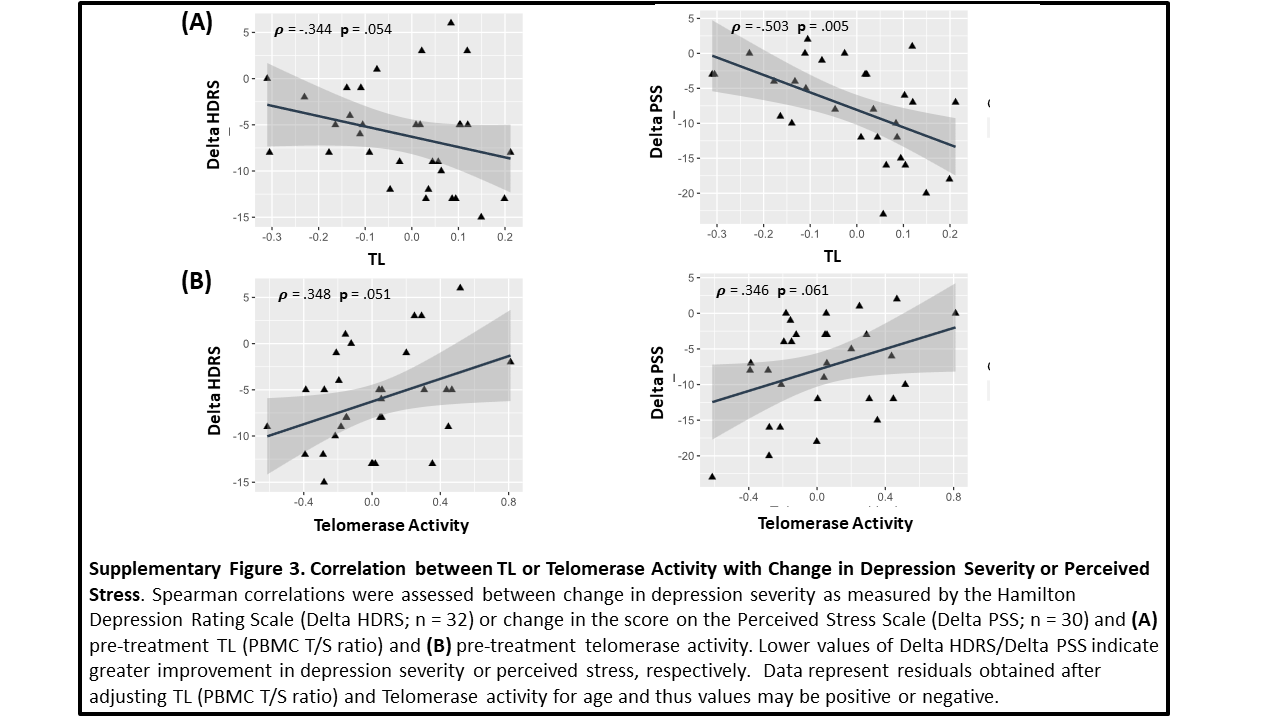
Supplementary Figure 3**

**
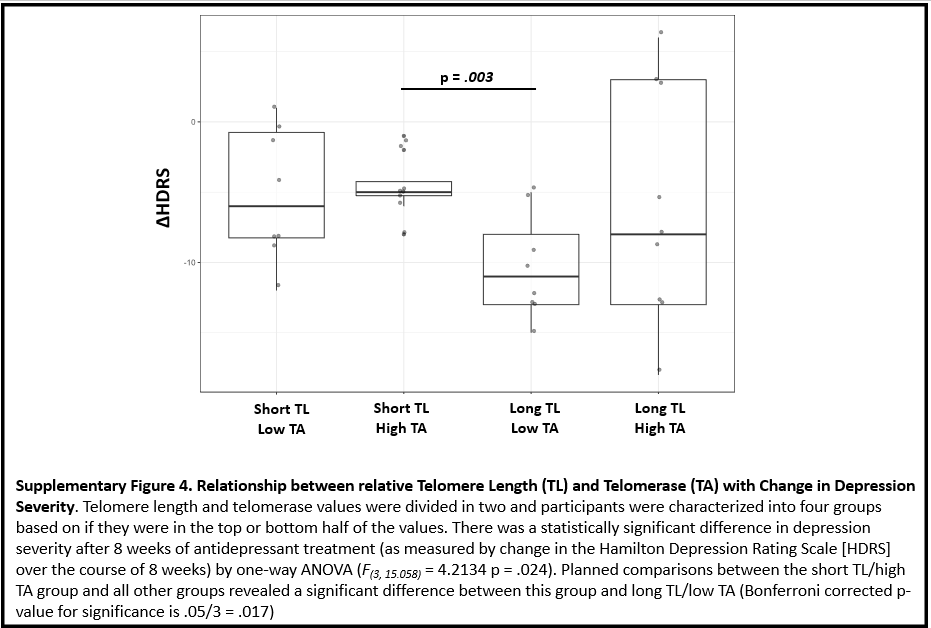
Supplementary Figure 4**

**
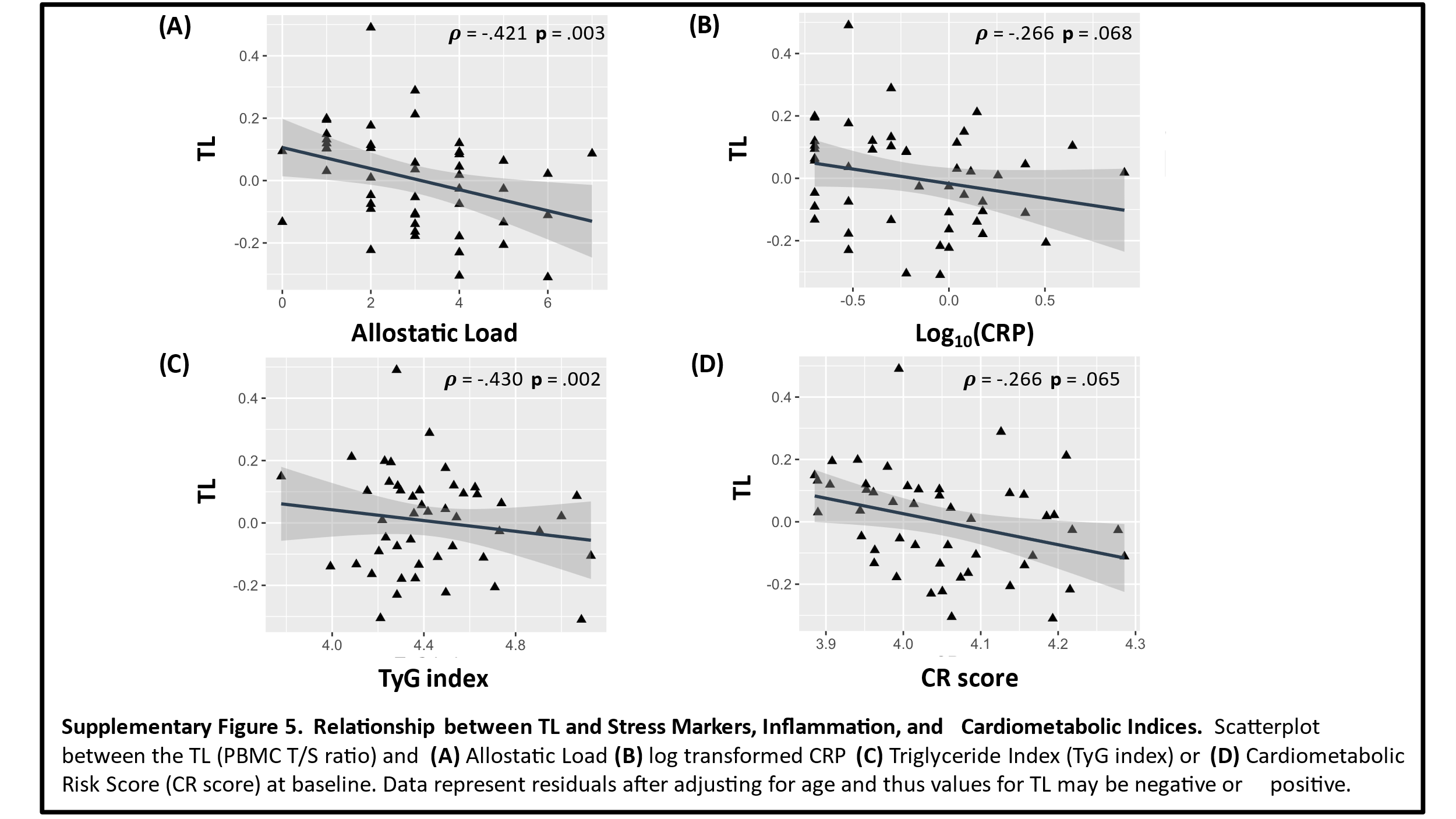
Supplementary Figure 5**

**Supplementary Table 1**

**Supplementary Table 2.**

**Supplementary Table 3**
